# Class II HDAC Inhibition Hampers Hepatic Stellate Cell Activation by Induction of MicroRNA-29

**DOI:** 10.1371/journal.pone.0055786

**Published:** 2013-01-31

**Authors:** Inge Mannaerts, Nathalie Eysackers, Oscar O. Onyema, Katrien Van Beneden, Sergio Valente, Antonello Mai, Margarete Odenthal, Leo A. van Grunsven

**Affiliations:** 1 Department of Cell Biology, Liver Cell Biology Lab, Vrije Universiteit Brussel, Brussels, Belgium; 2 Department of Human Anatomy, Liver Cell Biology Lab, Vrije Universiteit Brussel, Brussels, Belgium; 3 Istituto Pasteur - Fondazione Cenci Bolognetti, Dipartimento di Chimica e Tecnologie del Farmaco, Sapienza Universita' di Roma, Roma, Italy; 4 Institute of Pathology, University Hospital of Cologne, Cologne, Germany; University of Kansas Medical Center, United States of America

## Abstract

**Background:**

The conversion of a quiescent vitamin A storing hepatic stellate cell (HSC) to a matrix producing, contractile myofibroblast-like activated HSC is a key event in the onset of liver disease following injury of any aetiology. Previous studies have shown that class I histone deacetylases (HDACs) are involved in the phenotypical changes occurring during stellate cell activation in liver and pancreas.

**Aims:**

In the current study we investigate the role of class II HDACs during HSC activation.

**Methods:**

We characterized the expression of the class II HDACs freshly isolated mouse HSCs. We inhibited HDAC activity by selective pharmacological inhibition with MC1568, and by repressing class II HDAC gene expression using specific siRNAs.

**Results:**

Inhibition of HDAC activity leads to a strong reduction of HSC activation markers α-SMA, lysyl oxidase and collagens as well as an inhibition of cell proliferation. Knock down experiments showed that HDAC4 contributes to HSC activation by regulating lysyl oxidase expression. In addition, we observed a strong up regulation of miR-29, a well-known anti-fibrotic miR, upon treatment with MC1568. Our *in vivo* work suggests that a successful inhibition of class II HDACs could be promising for development of future anti-fibrotic compounds.

**Conclusions:**

In conclusion, the use of MC1568 has enabled us to identify a role for class II HDACs regulating miR-29 during HSC activation.

## Introduction

Fibrosis is characterized by excessive scar formation due to overproduction and deposition of extracellular matrix (ECM). This process usually occurs over a long period of time and can lead to organ dysfunction or death. There is no effective therapy available at the moment; therefore organ transplantation is often the only redress for patients with fibrosis. Donor shortage however underlines the need for more research on alternative therapies [1]. The identification of the hepatic stellate cells (HSCs) as the key cellular source of ECM synthesis in the liver was an important step towards the understanding of the mechanism of liver fibrosis and the development of new therapeutic strategies [2], [3]. Like liver sinusoidal endothelial cells and Kupffer cells, quiescent HSCs are non-parenchymal cells. They reside in the space of Disse and are lipid droplet containing cells that play a major role in the control and metabolism of retinol in the organism [4]. Following acute or chronic liver damage, these cells undergo a process of activation towards a myofibroblastic phenotype. This activation process is the result of a series of changes in gene expression [5]. The gene expression changes lead to a loss of retinoid containing lipid droplets, increased proliferation, motility, increased α-smooth muscle actin (α-SMA) expression, contractility and synthesis of extracellular components and matrix remodeling enzymes. This activation process is the dominant factor in liver fibrogenesis [2], [3]. As a consequence, inhibition of HSC activation can be an important target to develop new therapeutic strategies to intervene in liver fibrosis and cirrhosis [6], [7].

Alterations in the gene expression profile of HSCs during myofibroblastic activation are associated with changes in microRNA expression [8], [9]. microRNAs are small RNA molecules, that are able to inhibit protein synthesis by interacting with the 3′-untranslated region of mRNA derived from certain genes [10]. During HSC activation the expression of antifibrogenic microRNAs such as miR-29 is decreased [11], [12], whereas others like miR-21 are suggested to be increased [13]. Reduction of miRNA-29 levels during myofibroblastic transition of HSCs seems to play a predominant role for progression of fibrosis, because miRNA-29 was shown to inhibit collagen synthesis and profibrotic growth [11], [12] .

In addition to microRNA alterations during myofibroblastic HSC activation, recent studies have shown the importance of epigenetic regulation underlying the transdifferentiation of HSCs *in vitro*. The group of Mann has demonstrated that inhibition of DNA methylation by 5-aza-2-deoxycytidine prevents activation of the stellate cells [14]. Additionally, histone deacetylase (HDAC) inhibitors, trichostatin A and valproic acid have been identified as potent inhibitors of HSC activation both *in vitro* and *in vivo*. Effects on collagen deposition, actin filament formation and HSC proliferation have been observed [15], [16], [17], [18]. HDACs catalyse the removal of acetyl groups from histone proteins, thereby inducing a positive charge on the lysine side chains of histones H3 and H4 and preventing the access of transcriptional complexes to DNA. Generally HDAC activity is linked to transcriptional repression. Four families of HDACs are characterized since the first identification of HDACs in 1996: the class I, II and IV HDACs and the class III or SIRT family. Evidence is accumulating that class I is directly involved in regulation of cell growth and death, whereas class II members are expressed in a tissue-specific manner and regulate differentiation processes, such as muscle- and neuronal differentiation. Class II HDACs are divided in two sub classes, class IIa and class IIb. Class IIa comprises HDACs 4, 5, 7 and 9, whilst class IIb HDACs are HDAC6 and 10 that are characterized by the presence of two catalytic domains. Another major difference between class I and class II HDACs is that while class I HDACs reside in the nucleus, due to the presence of a nuclear export signal, class IIa HDACs can shuttle between the nucleus and the cytoplasm [19]. The class IIb HDAC6 displays distinct functionality, as its inhibition stimulates tubulin acetylation and influences cell motility [20], [21], [22].

HDAC inhibitors are widely used for the treatment of cancer and in general use of these compounds is well tolerated by the majority of patients. Efforts are made to generate HDAC inhibitors with specificity towards one class or individual HDACs. Recently, an inhibitor with specificity towards class II HDACs, MC1568, was developed [23], [24], [25], [26]. So far, the role of class II HDACs during stellate cell activation has not been addressed. Nevertheless, if this inhibitor has the potential to hamper hepatic stellate cell activation, a class II-selective HDAC inhibitor would be preferred over pan-HDAC inhibitors because class II HDACs are generally less involved in control of transcription and less toxic. In particular, MC1568 was shown devoid of cell cycle and apoptotic effects in diverse human cancer cell lines [23], [27], [28]. Here, we show that administration of the selective class II HDAC inhibitor MC1568, results in a marked decrease in stellate cell activation *in vitro*. Daily treatment inhibits HSC proliferation, collagen secretion and the differentiation of the HSC toward a more myofibroblast-like cell, and this through the induction of microRNA-29.

## Materials and Methods

### Isolation, culture and treatment of HSCs

The study protocol was approved by the Institutional Animal Care and Use Committee of Vrije Universiteit Brussel, permit number 10-212-3, and National Institutes of Health principles of laboratory animal care (NIH publication 86-23, revised 1995) were followed.

Mouse HSCs were isolated from normal livers of male Balbc mice (25–35 g) as previously described [29]. After isolation, cells were plated for several days. A class II-selective inhibitor 3-{4-[3-(3-fluorophenyl)-3-oxo-1-propen-1-yl]-1-methyl-1H-pyrrol-2-yl}-N-hydroxy-2-propenamide (MC1568), dissolved in DMSO was administered daily in an optimized concentration of 1 µM. For *in vivo* HSC activation mice underwent 8 intraperitoneal injections, over 4 weeks, of 50 µl CCl_4_/100 g body weight in mineral oil (Sigma-Aldrich, St. Louis, MO, USA). To study the therapeutic effect of MC1568 *in vivo*, mice received intraperitoneal injections of the inhibitor (MC1568, 50 mg/kg) [24], [26] every two days, after initiation of liver injury by 2 weeks of CCl_4_ treatment.

### Picrosirius Red Staining

Twelve-µm frozen sections were air-dried, fixed with SUSA's fixative for 1 hour and stained for 45 minutes with 0.1% Sirius Red F3BA in a saturated picric acid solution. For each condition, 21 pictures were made using an Axioskop light microscope (Carl Zeiss, Zaventem, Belgium) and the pictures were recorded using an Axiom digital camera. Red staining was quantified using NIH ImageJ software (http://rsb.info.nih.gov/ij/).

### qPCR

RNA from cells and total tissue was isolated respectively with the Gene Jet RNA purification kit (Fermentas, St. Leon-Rot, Germany) and trizol (Invitrogen, Eugene, Oregon, USA) and was reverse-transcribed using the RevertAid™ Premium Reverse Transcriptase kit (Fermentas). For real-time polymerase chain reaction (PCR), GoTaq qPCR Master Mix with BRYTE green was used (Promega, Madison WI, USA), subjected to quantitative PCR (qPCR) in an ABI 7500 Real Time PCR System, and analyzed using System SDS software (Applied Biosystems). The gene-specific primers we used are listed in table 1. The optimal housekeeping gene was determined using Normfinder [30] and Bestkeeper [31] software. For *in vitro* assays, GAPDH was used as reference gene, while for analysis of qPCR data on total liver RNA was normalized with HPRT1. The fold change differences were determined using the comparative threshold cycle method. Similarly, for microRNAs, total RNA from Trizol extractions was subjected to reverse transcription using the miScript II Reverse transcriptase kit (Qiagen, Hilden, Germany). The cDNA was then used for qPCR analysis using microRNA specific primers (listed in table 1), a universal primer (Qiagen) and GoTaq qPCR Master Mix with BRYTE green (Promega). The Ct-values of detected microRNAs were corrected for the input of microRNA by subtracting the Ct-values of RNU6 endogenous control microRNA. Again the deltadelta-Ct method was used for calculation of the fold change differences.

**Table 1:** List of QPCR primers.

*Gene*	*Genebank Accession number*	*Primers sequence (Forward/Reverse)*
*Gapdh*	NM_008084.2	5′-cctgcttcaccaccttcttg -3′/5′-tgtccgtcgtggatctgac -3′
*Hdac4*	BC066052	5′-aaccactcaactcatcttgtagctt-3′/5′-ccccactaaggttcacaagg-3′
*Hdac5*	AF207748	5′-tctgtcaccgccagatgtt-3′/5′-atggcggtcaagtcatgg-3′
*Hdac6*	BC041105	5′-ccatgaagcctctgaacacc-3′/5′-atggcggtcaagtcatgg-3′
*Hdac7*	BC057332	5′-ccatgggggatcctgagt-3′/5′-gcaaactctcgggcaatg-3′
*Hdac9*	BC098187	5′-tggaacactgttgaaggtagtctg-3′/5′-gataaccacaacatttcaacttaacaa-3′
*Hdac10*	BC064018	5′-gctcgctggaaattgctg-3′/5′-gcgactggcaatcactgtta-3′
*Acta 2*	NM_007392	5′-ccagcaccatgaagatcaag-3′/5′-tggaaggtagacagcgaagc-3′
*Col1a1*	NM_007742	5′-cctaagggtaccgctgga-3′/5′-tccagcttctccatctttgc-3′
*Lox*	NM_010728	5′-tcactgcgctcgttctgat-3′/5′-cgatcgaaagtatgagggatg-3′
*Hprt1*	NM_013556	5′-tcctcctcagaccgctttt-3′/5′-cctggttcatcatcgctaatc-3′

*microRNA*	*Mirbase Accession number*	*Primer sequence*
*RNU6*	Not available	acgcaaattcgtgaagcgtt
*miR-29a*	MIMAT0000535	tagcaccatctgaaatcggtta
*miR-29b*	MIMAT0000127	tagcaccatttgaaatcagtgtt
*miR-29c*	MIMAT0000536	tagcaccatttgaaatcggtta

### Western Blotting

Cells were washed twice with ice-cold phosphate-buffered saline and incubated for 20 min on ice with lysis buffer (170 mM NaCl, 10 mM EDTA, 50 mM Tris pH 7.4, 50 mM NaF, 0.2 mM dithiothreitol and 0.5% NP-40) supplemented with protease and phosphatase inhibitors. The protein concentration was measured using a bicinchoninic acid (BCA) determination kit (Pierce Chemical Co., Rockford, IL, USA). Ten or twenty microgram of protein was separated on 6–10% Tris–glycine SDS-Polyacrylamide gels and transferred onto polyvinyldifluoride (PVDF) membranes (Amersham Biosciences, Little Chalfont, UK) using a semidry blotting apparatus (Apollo™, Continental Lab Products, San Diego, CA, USA). For western blot analysis, the membrane was blocked in 5% milk in TBS-Tween. After overnight incubation with primary antibodies (Information on suppliers and working concentrations in table 2) and 1 hour incubation with horseradish peroxidase conjugated secondary antibodies (1/20000) (Dako, Glostrup, Denmark), proteins were visualized with the ECL chemiluminescence detection system (Pierce Chemical Co.).

**Table 2:** List of antibodies for immunocytochemistry and western blot.

Protein	Supplier	Application	Used concentration
α-Smooth muscle actin	Sigma	WB	1/1000
Acetylated Tubulin	Sigma	ICC	1/500
β-actin	Sigma	WB	1/1000
Collagen I	Abcam	WB	1/500
Hdac4 (N18)	Santa Cruz	WB	1/500
Lysyl oxidase	Santa Cruz	WB	1/250

### Cell Proliferation assay

Cell proliferation was measured as active DNA synthesis with the Click-iT EdU Cell Proliferation Assay Kit (Invitrogen, Eugene, OR). 3750 cells per cm^2^ were plated in presence or absence of 1 µM MC1568, after 48 hours EdU labeling was initiated, and another 48 hours later (on day 4), cells were formalin fixed. Visualization of the EdU incorporation was obtained according to manufacturer's instructions. The ratio of total cells and EdU incorporated cells was calculated.

### Immunocytochemistry

Cells were formalin-fixed after 4 days of culture in the presence or absence of 1 µM MC1568. Following permeabilization with 0.1% Triton-X 100 (in PBS with 1% bovine serum albumin) cells were overnight incubated with primary antibody (acetyl-tubulin; 1/1,000, Sigma). Antibody binding was visualized using Alexa488-labeled antibody (1/200). Images were taken with an AxioCam MRc 5 digital camera (Carl Zeiss, Zaventem, Belgium).

### Small Interfering RNA Transfection

All small interfering RNAs (siRNAs) used in this study were siRNA stealths from Invitrogen; HDAC4: MSS209678, MSS209679, HDAC5: MSS247268, MSS247269, HDAC6: MSS205076, MSS205078 (Invitrogen). siRNAs (5 nM) were transfected using Rnai MAX Transfection Reagent (Invitrogen) according to the manufacturer's instructions. Cells were transfected twice over 5 days and collected 4 days after the final transfection. A nonsilencing siRNA was used as a control.

### Liver Enzymes

Blood samples were taken from the inferior vena cava, centrifuged at 2,000 g for 10 minutes, and stored at −20°C. Serum aspartate aminotransferase (AST) and alanine aminotransferase (ALT) activities were determined at 37°C with an automated analyzer Spotchem EZ (Menarini diagnostics Benelux n.v. Valkenswaard, the Netherlands).

### HDAC activity assay

HDAC activity was measured in total liver protein using the HDAC-Glo Screening Assay (Promega). Some adaptations were made to the manufacturer's protocol for the use on total tissue. In brief 10 µg of protein was diluted in HDAC-Glo buffer. 100 µl of assay mix, containing the substrate and the developer reagent, and diluted sample (1∶1) were transferred to 96-wells and after 30 min incubation at room temperature, luminescence was measured.

### Statistical analysis

Results are presented as the mean + SEM. Data were subjected to analysis of variance (ANOVA) followed by Tukey's post-test. Statistical analysis of values for comparison between 2 groups was performed using two tailed Student t-test. A Pearson correlation test was performed to determine the correlation between HDAC-activity and Sirius Red staining. *** P<0.001, ** 0.001<P<0.01, * 0.01<P<0.05

## Results

### Expression pattern of Class II HDACs during hepatic stellate cell activation

**Figure 1:**
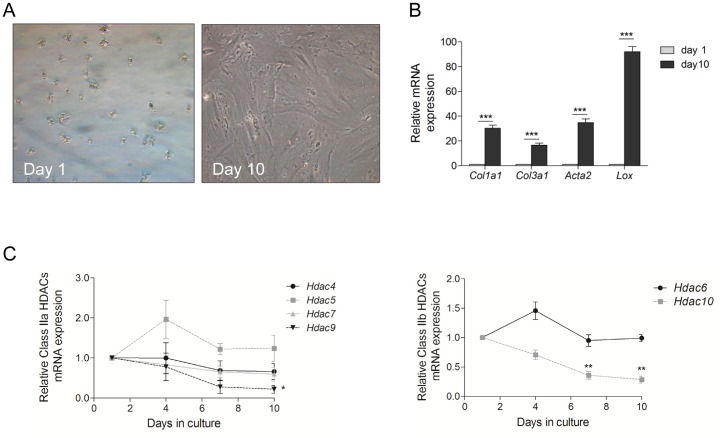
Class IIa and class IIb HDACs are constantly expressed during *in vitro* mouse hepatic stellate cell activation. Mouse HSCs were isolated from healthy mice and cultured for the indicated time. (A) Shows morphological changes associated to the mHSC activation process *in vitro*. (B) mRNA levels of activation markers *Col1a1*, *Col3a1*, *Acta2 and Lox* were determined using qPCR to confirm *in vitro* activation. (C) mRNA expression of Class IIa and IIb *Hdac*s was measured by qPCR. Values represent 3 replicates, ***: p<0,001, **: p<0, 01, *: p<0, 05.

Previously, we have shown that class I HDACs play an important role during HSC activation, but selective inhibition of this group of enzymes could however not completely block the transdifferentiation towards myofibroblast-like cells [18]. In this current work, class II HDACs expression was tested by qPCR in freshly isolated and culture activated HSCs. Primary mouse HSCs were culture activated, leading to the typical morphological changes from a star shaped cell containing many lipid droplets at day 1 towards a myofibroblast like cell at day 10 (Figure 1A). This *in vitro* activation is further confirmed by the increased *Acta2*, *Col1a1*, *Col3a1* and *Lox* (encoding for α-SMA, collagens type I and III and lysyl oxidase) mRNA expression between day 1 and day 10 during differentiation from quiescent to activated HSCs (Figure 1B). In both quiescent and activated cells, all class II HDACs are expressed. During HDAC activation most class II HDACs are constantly expressed, however a significant down-regulation after 10 days of culture was observed for *Hdac9* and *Hdac10* (Figure 1C).

### Inhibition of Class II HDAC activity hampers HSC activation

**Figure 2:**
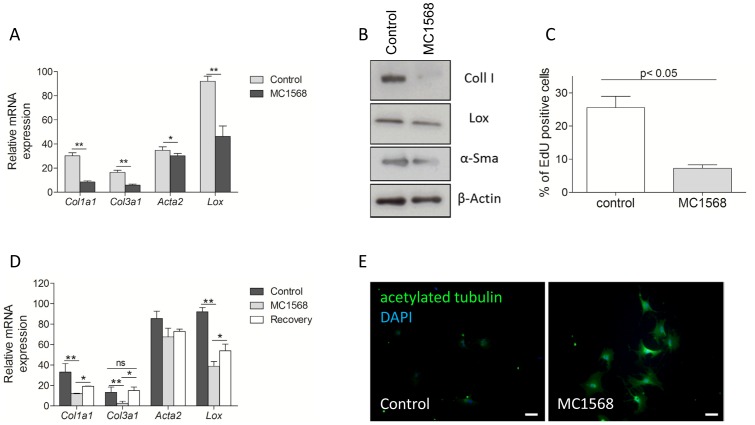
Effect of MC1568 treatment on stellate cell activation markers. (A) Freshly isolated HSCs were plated in presence or absence of 1 µM MC1568 for 10 days. The effect on HSC activation markers *Acta2*, *Lox*, *Col1a1* and *Col3a1* was evaluated by qPCR. (B) Mouse HSCs were cultured for 10 days in presence or absence of 1 µM MC1568. The effect on HSC activation markers Collagen I, Lysyl oxidase and α-Smooth muscle actin was investigated by western blot. β-actin was used as a loading control. (D) The effect of MC1568 treatment on HSC proliferation was investigated with an EdU incorporation assay. Cells were cultured for 48 hours in the presence or absence of MC1568. Nuclei were stained with 4′,6-Diamidino-2-phenylindole. The percentage of EdU-positive cells was determined from three independent experiments. (D) In order to test the reversibility of MC1568 treatment, freshly isolated mouse HSCs were treated for 7 days, after 7 days the inhibitor was washed out and cells were further cultured until day 10 (recovery). Then cells were collected and mRNA expression of HSC activation markers was determined. (E) Freshly isolated mouse HSCs were cultured for seven days. At day seven, cells were formalin fixed and stained with an antibody against the acetylated form of tubulin. Prior to fixation, cells were treated with 1 µM MC1568 for 24 hours. Nuclei were visualized with 4′,6-Diamidino-2-phenylindole. Scale bar = 100 µM. * p<0.05, ** p<0,01.

To investigate whether class II HDACs could play a role during HSC activation, we incubated freshly isolated mouse HSCs with increasing concentrations of the class II selective HDAC inhibitor MC1568 [24]. This inhibitor has been extensively investigated in cancerous and non-cancerous cell types and showed effective inhibition of class II HDACs without showing toxicity [26], [28], [32], [33]. We observed no clear difference in morphological appearance of HSCs treated with 1 µM MC1568, but gene expression levels of several genes known to be regulated during HSC activation *in vitro* and *in vivo* were analyzed during a 10-day *in vitro* culture period using qPCR. The strongest MC1568-dependent gene expression changes during HSC activation were observed for *Col1a1*, *Col3a1*, *Acta2* and *Lox* (Figure 2A). At the protein level, MC1568 treatment clearly inhibited collagen I expression, while the effect on lysyl oxidase and α-SMA was more moderate (Figure 2B). Increased proliferation, a characteristic of transdifferentiating HSCs, was investigated using an EdU incorporation assay. Proliferation was greatly reduced in the MC1568-treated HSCs when compared with control HSCs (Figure 2C). We performed a wash-out experiment to demonstrate the reversibility of MC1568-treatment. Hereto, HSCs were cultured in the presence of MC1568 for 7 days, then the inhibitor was washed out and cells were cultured for three more days (recovery). An up-regulation of *Lox* and *Coll3a1* mRNA was observed, until a level that was not significantly different from the control (*Coll3a1*). The mRNA expression levels of *Acta2* and *Col1a1* recovered only modestly (Figure 2D). When the cells were stained for acetylated tubulin proteins, we observed a clear increase in acetylated tubulin in the MC1568-treated HSCs when compared with control cells (Figure 2E), a clear indication of a decreased class II HDAC activity.

### MC1568 effect on carbon tetrachloride induced liver fibrosis

**Figure 3:**
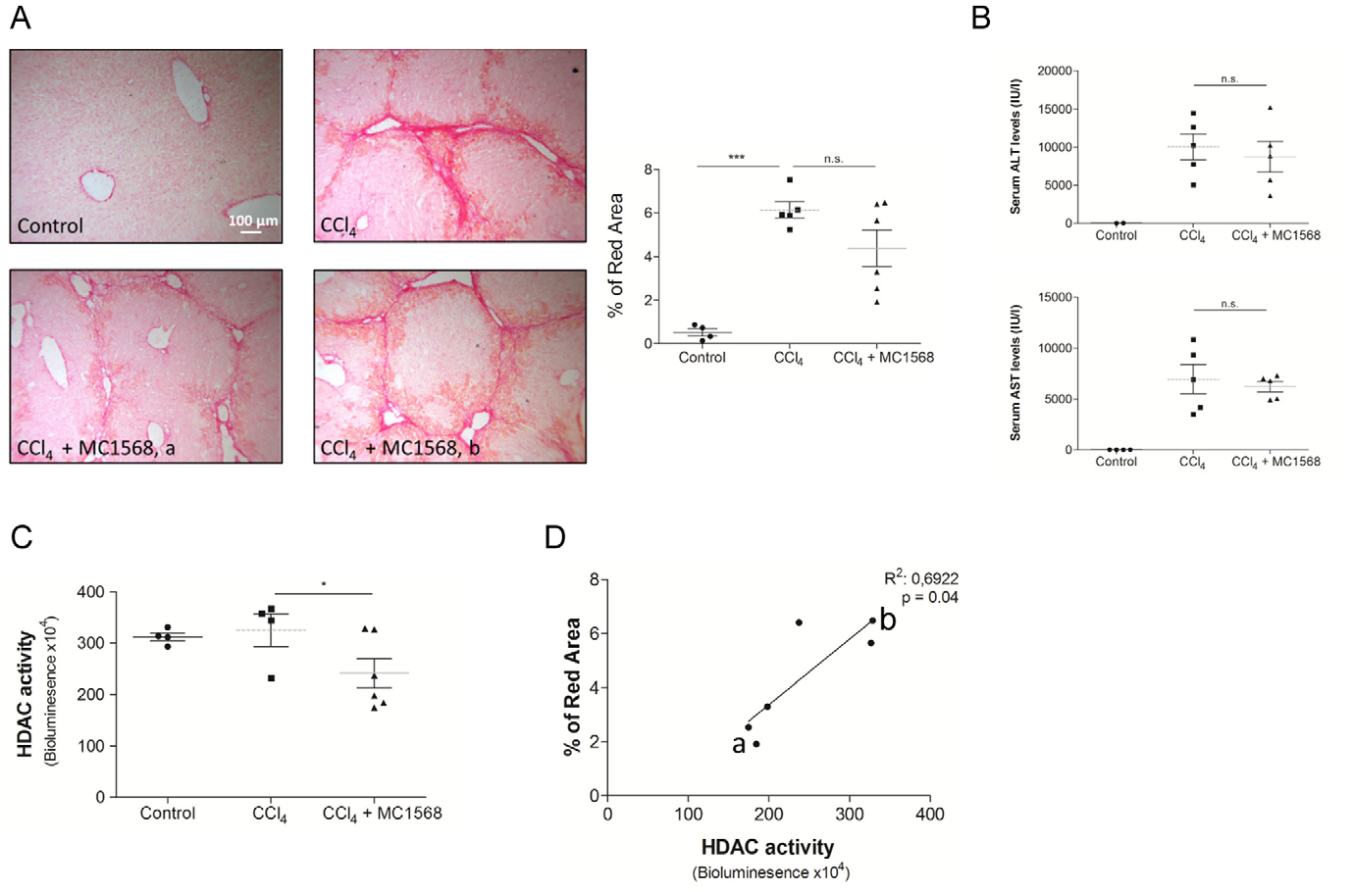
Effect of MC1568 treatment on fibrogenesis in a CCl_4_ induced fibrosis mouse model. Mice were CCl_4_ treated twice a week for a total period of 4 weeks. After the second week, mice also received intraperitoneal injections with MC1568 (50 mg/kg) every two days for two more weeks. The day after the final CCl_4_ injection, mice were sacrificed and liver tissue was extracted for analysis. (A) Sirius Red staining was performed to visualize deposited collagens. Image J software was used for quantification of the red surface area. Scale bar = 100 µm. (B) Serum levels of ALT and AST. (C) HDAC activity was measured in protein lysates of livers at the end of treatments using HDAC-Glo. (D) A mathematical correlation between the HDAC activity and the red stained area, after Sirius staining was determined using Pearson correlation method. The a and b refer to HDAC-activity values of the corresponding liver lysates of the images given for CCl_4_ + MC1568 in A).

Beneficial effects of HDAC-inhibitor treatment in mouse models of fibrosis in liver, heart and kidney have been reported [18], [34], [35], [36], [37]. We addressed the function of class II HDACs during liver fibrosis by the use of MC1568 in a CCl_4_ mouse model for liver fibrosis. Mice were treated for 2 weeks with CCl_4_, followed by two more weeks of co-treatment of CCl_4_ and MC1568 or DMSO. The MC1568 concentration used and way of administration was described before [24], [26]. During the course of our treatment the overall appearance of the mice was normal; the MC1568 therapy did not influence their behavior, liver- or body -weight and ALT/AST serum concentrations showed no significant difference (Figure 3B). While we had the impression that in some mice MC1568 treatment had a beneficial effect, only a minor reduction (statistically not significant) of red surface area was observed in the group treated with both CCl_4_ and MC1568 (CCl_4_+MC1568) compared to the animals treated for 4 weeks with CCl_4_ alone (Figure 3A). However, we noted a strong variation between the mice within the CCl_4_+MC1568 group. In order to get more insight in this, a HDAC-activity assay was performed. This assay allowed us to measure the enzyme activity in protein extracted from liver samples after treatment of mice with either CCl_4_ alone or a combination of CCl_4_ and MC1568. Treatment of mice with MC1568 successfully hampered HDAC activity in the liver (Figure 3C). Again some disparity between animals was observed, but a correlation analysis of individual mice shows a relationship between reduced HDAC-activity and less collagen deposition (Figure 3D). This correlation suggests that an optimized class II HDAC inhibition in HSCs *in vivo* could be a promising anti-fibrotic strategy.

### Class II HDAC knock-down partially hinders HSC activation through induction of microRNA-29

**Figure 4:**
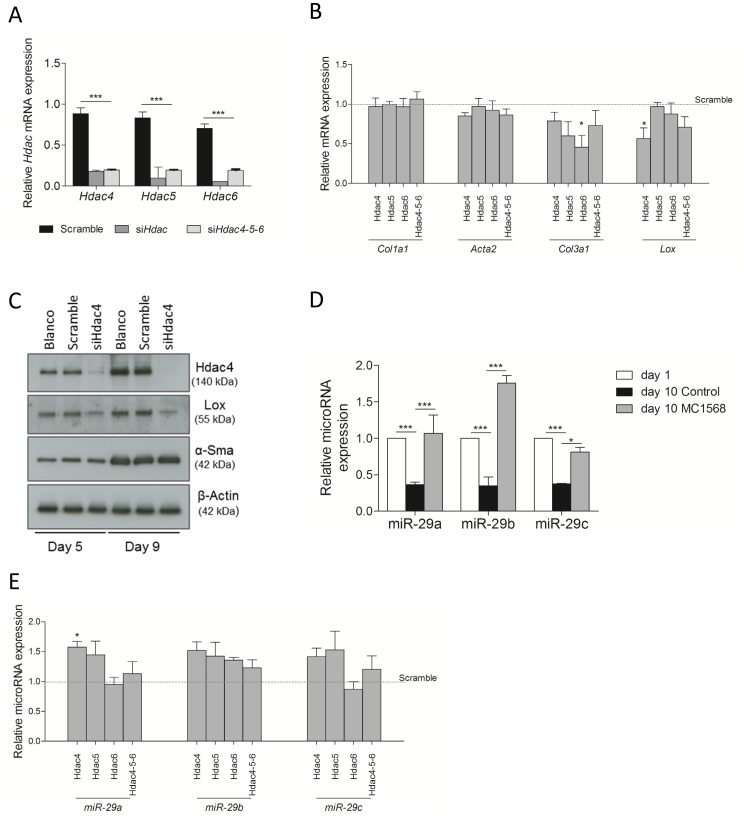
Role of class II HDACs during HSC activation by selective siRNA mediated knock-down. Freshly isolated HSCs were transfected with siRNA after 24 hours of culture and a second time on the fifth day of culture, RNA was collected 3 days after the final transfection (day 8). (A) *Hdac* knock-down was evaluated by qPCR. (B) In addition the effect on activation markers was determined by qPCR. The dashed line represents the gene expression level in cells transfected with non-specific siRNA. (C) At day 4 after the second transfection protein samples were harvested. The effect of HDAC knock-down on Lox and expression was confirmed by WB. β-actin was used as loading control. (D) The effect of HDAc inhibition by MC1568 treatment was investigated after 10 days of treatment in culture. Total RNA was isolated from cultured cells, a microRNA reverse transcription was performed followed by qPCR assay for selective amplification of miR-29a, miR-29b and miR-29c, and RNU6 was used as internal control. (E) Freshly isolated HSCs were transfected with siRNA after 24 hours of culture and a second time on the fifth day of culture, RNA was collected 3 days after the final transfection (day 8). Total RNA was isolated from cultured cells and a qPCR assay for selective amplification of miR-29a, miR-29b and miR-29c was performed, RNU6 was used as internal control. miR-29 expression in HDAC knock down samples was calculated relative to miR-29 expression in a sample transfected with non-targeting siRNA (represented by dashed line). Graphs are representative of at least three experiments. *P<0.05, **P<0.01, ***P<0.001.

To further confirm the contribution of class II HDACs to HSC activation and to gain insight in the mechanisms involved in the effect of MC1568-treatment on HSC transdifferentiation, we determined the effect of selective knock-down of the individual HDACs on HSC activation. siRNAs against HDACs shown to be inhibited by MC1568, *i.e*., Hdac4, Hdac5 and Hdac6 [24] were used individually (Figure 4A, siHdac) and combined and the effect on activation marker expression was tested. qPCR analysis for *Col1a1*, *Acta2*, *Col3a1* and *Lox* was performed on freshly isolated HSCs where Hdac4,5 and 6 expression was silenced using siRNA. Col3a1 and Lox mRNA levels were affected by class II HDAC knock-down, while no effect on Col1a1 or Acta2 was observed (Figure 4B). Only knock-down of HDAC4 reduced LOX protein levels (Figure 4C), knock-down of others did not influence significantly protein levels of collagen I or lysyl oxidase (data not shown). Recent studies have investigated the role of microRNA-29 family members in the regulation of collagen expression during HSC activation, both, *in vitro* and *in vivo* [11], [12], [38]. To investigate this level of regulation in HSCs, freshly isolated mHSCs were either treated with 1 µM of MC1568 or DMSO. In the cells with inhibited HDAC-activity, miR-29 expression was strongly induced, while during normal culture the expression of miR-29a, -29b and -29c were effectively inhibited compared to the day 1 control as was described before by others (Figure 4D) [11], [12]. This suggests that class II HDACs could affect collagen expression in HSCs through up-regulation of miR-29, as could be confirmed by siRNA-mediated knock-down of class II HDACs 4 and 5 (to a lesser extend HDAC6) induced miR-29 expression (Figure 4E).

## Discussion

HDACs regulate a variety of physiological and pathophysiological processes. Previously, it was reported that HDACs play a role in fibrotic disorders [36], [39]. Earlier, we have demonstrated a task for class I HDACs during fibrogenesis and stellate cell activation. However, in this study it was clear that chemical inhibition of class I HDAC activity was not sufficient to block HSC activation and fibrosis completely [18]. In this study, we show for the first time a role for class II HDACs during HSC activation.

The role of HDACs in cell proliferation is well known, with mostly class I HDACs contributing to this process as indicated by the up regulation of p21 [40], [41]. This could be an indication that the effect on HSC proliferation observed upon MC1568 treatment may not be direct, but rather a consequence of hampered HSC activation. Previous research has proven the selectivity of MC1568 towards HDACs 4, 5 and 6, we therefore used siRNA mediated knock down of these 3 HDACs individually and combined to study their role in HSC activation. An inhibitory effect of HDAC4 knock down was observed on Lox gene and protein expression, suggesting that HDAC4 plays an important role in the regulation of collagen cross linking by affecting Lox, an enzyme that regulates the cross linking of elastin and collagen. Cross-linking of the collagen increases matrix stiffness and induces myofibroblast differentiation *in vitro*. It was hypothesized that due to the excessive matrix deposition in the liver, liver stiffness would increase and promote the maintenance of the activated HSC phenotype. Treatment of rats with CCl_4_ and a Lox inhibitor BAPN was shown to effectively reduce the number of αSMA positive cells. Counter intuitively, no effects on collagen deposition were observed. It was suggested that since HSC activation was inhibited, other cellular sources were responsible for early matrix deposition [42], [43]. Similar to previously described observations in skin fibroblasts [44], [45], HDAC4 also plays a role in TGFβ-induced myofibroblast differentiation of HSCs. However, in our study no effect on *Acta2* was observed, as was reported in the skin fibroblast model. This clearly indicates that one cannot compare the myofibroblast differentiation of skin fibroblasts with the transdifferentiation of quiescent HSCs, although both processes can be induced by TGFβ. We did however observe a reduced up regulation of Lox upon TGFβ administration to quiescent HSCs in HDAC4 knock-down conditions (data not shown). Our results point towards HDAC4 as an interesting target for inhibition of *in vivo* fibrogenesis, corroborating the findings of a recent study showing an HDAC4-dependent regulation of matrix metalloproteases [46]. An increased matrix metalloprotease activity and decreased matrix cross-linking, would result in a less stable matrix that is more sensitive to degradation, not favoring the maintenance of the activated HSC phenotype.

Acetylation of lysine residues modulates protein-histone and histone-DNA interactions and thereby regulates many cellular processes. The acetylation status is the result of balanced activity of histone acetyltransferases and deacetylases, and a deregulation of this balance by aberrant HDAC activity is linked to various diseases, including cancer, neurological, inflammatory and fibrotic disorders. Recent studies show that not only gene expression is regulated by HDACs, but also microRNA expression can rapidly be altered upon HDAC-inhibition. For example, in chronic lymphocytic leukemia, overexpressed HDACs block critical miRs resulting in pro-survival signals [47]. In addition, there is increasing interest in the role of miRs during the HSC activation process. Although some inconsistencies exist between different reports, it becomes clear that members of the miR-29 family are important regulators of collagen expression during HSC transdifferentiation [11], [12]. However, not much is known on how these miRs are regulated in the liver. As we observed a strong and reproducible effect on collagen expression (both type I and type III) upon HDAC inhibition using MC1568 or siRNA, we investigated the impact of MC1568 treatment on miR-29 expression. We could show that in freshly isolated mouse HSCs microRNA-29a, b and c are regulated by class II HDACs, underscoring the potential of HDAC inhibitors in therapeutic intervention of fibrosis. These results are in line with reports in chronic lymphocytic leukemia patients showing that microRNA-29 (miR-29) expression can be regulated by HDAC-activity [48].

In the CCl_4_-induced liver injury mouse model, we did not observe the expected effects on collagen deposition and other key fibrosis markers (Figure 4). Strong variations between the different mice within one condition, made the interpretation of the results rather difficult. This is possibly due to an ineffective delivery or the fast metabolization of MC1568. An HDAC activity assay on protein lysates of total liver after CCl_4_ and MC1568 treatment enabled us to correlate the degree of HDAC inhibition with the collagen deposition measured by Sirius red staining. This suggests that the MC1568 delivery to the liver is not optimal. Previous reports describing MC1568 dependent effects on a variety of physiological processes have described organ selective effects. No effect on the liver was described in these studies [24], [26]. The use of selective delivery approaches with HSC-targeting liposomes [49], [50] could improve the delivery of the compound to HSCs.

Ever since the contribution of HSCs to matrix deposition and liver fibrosis was discovered, attempts to inhibit their activation have been made. Evidence for anti-fibrotic properties of drugs affecting HSC activation is mostly limited to *in vitro* data [14], [51], [52], [53], [54], [55], [56], [57], [58]. Our experience with MC1568 shows that the *in vitro* anti-fibrotic potential of a given compound cannot always be translated to *in vivo* benefits due to impending problems in stability, turnover or delivery to the HSCs. The use of compounds with a clinical history (non-liver) and information about half-life and side effects will largely circumvent this problem when evaluating their anti-fibrotic properties. Nevertheless, the use of MC1568 has enabled us to identify a role for class II HDACs during HSC activation *in vivo*; however, its clinical potential as an anti-fibrotic needs further development. We also conclude that the anti-fibrotic effect of class II HDAC inhibition is partially the result of subsequent regulation of miR-29 and collagen expression.
